# Heparanase is the possible link between monkeypox and Covid-19: robust candidature in the mystic and present perspective

**DOI:** 10.1186/s13568-023-01517-y

**Published:** 2023-01-27

**Authors:** Hayder M. Al-Kuraishy, Ali I. Al-Gareeb, ‏Helal F. Hetta, Athanasios Alexiou, Marios Papadakis, Gaber El-Saber Batiha

**Affiliations:** 1 Department of Clinical Pharmacology and Therapeutic Medicine, College of Medicine, ALmustansiriyiah University, Baghdad, Iraq; 2 Department of Medical Microbiology and Immunology, Faculty of Medicine, Assiut University, Assiut, 71515 Egypt; 3 Department of Science and Engineering, Novel Global Community Educational Foundation, Hebersham, NSW 2770 Australia; 4 AFNP Med, 1030 Vienna, Austria; 5 Department of Surgery II, University Hospital Witten-Herdecke, University of Witten-Herdecke, Heusnerstrasse 40, 42283 Wuppertal, Germany; 6 Department of Pharmacology and Therapeutics, Faculty of Veterinary Medicine, Damanhour University, AlBeheira, Damanhour, 22511 Egypt

**Keywords:** Heparanase, SARS-CoV-2, Monkeypox

## Abstract

Heparanase (HPSE) is an endoglycosidase cleaves heparan sulfate (HS) and this contributes to the degradation and remodeling of the extracellular matrix. HS cleaved by HPSE induces activation of autophagy and formation of autophagosommes which facilitate binding of HPSE to the HS and subsequent release of growth factors. The interaction between HPSE and HS triggers releases of chemokines and cytokines which affect inflammatory response and cell signaling pathways with development of hyperinflammation, cytokine storm (CS) and coagulopathy. HPSE expression is induced by both SARS-CoV-2 and monkeypox virus (MPXV) leading to induction release of pro-inflammatory cytokines, endothelial dysfunction and thrombotic events. Co-infection of MPX with SARS-CoV-2 may occur as we facing many outbreaks of MPX cases during Covid-19 pandemic. Therefore, targeting of HPSE by specific inhibitors may reduce the risk of complications in both SARS-CoV-2 and MPXV infections. Taken together, HPSE could be a potential link between MPX with SARS-CoV-2 in Covid-19 era.

## Introduction

Heparanase (HPSE) is an endoglycosidase cleaves heparan sulfate (HS) and this contributes to the degradation and remodeling of the extracellular matrix. HPSE is synthesized as 68 kDa in the endoplasmic reticulum. In the Goli apparatus, HPSE is processed into proHPSE (65 kDa) and the proHPSE is secreted into the extracellular space (Fux et al. 2009).

Outside the cell, HPSE binds different molecules including HS and mannose-6-phosphate (low density lipoprotein receptor related protein) leading to endocytosis and transport of these molecules. Within the lysosome and by action of L-cathepsin the 6 kDa subunit is cleaved with formation of heterodimer form of HPSE (Vlodavsky et al. 2012). HPSE induces activation of autophagy and formation of autophagosommes which facilitate binding of HPSE to the HS and subsequent release of growth factors like epidermal growth factor (EGF) and vascular endothelial growth factor (VEGF) (Jayatilleke and Hulett 2020). The interaction between HPSE and HS triggers releases of chemokines and cytokines which affect inflammatory response and cell signaling pathways (Jayatilleke and Hulett 2020). HPSE is involved in progression of many diseases including viral infections and cancer. Expression of HPSE is link with propagation of angiogenesis, metastasis and enhancement of tumor progression (Masola et al. 2018).

HS is consisting of a core protein and glycosaminoglycan (GAG), like syndecan and perlecan. HS proteoglycans are mainly expressed on the cell membrane (Masola et al. 2018; Qiao et al. 2020). HS is degraded by HPSE, and HS promotes cell adhesion and motility as well; it binds and prevents degradation of chemokines, cytokines, morphogens and growth factors (Qiao et al. 2020). Of note, HS acts as an endocytosis receptor regulates degradation of extracellular molecules, and increasing trans-endothelial transmission of chemokines. Through proteolytic shedding of perlecan and syndecan, HS promotes regulation of intracellular stress which maintains development of stem cell (Zhang et al. 2014).

**Fig. 1:**
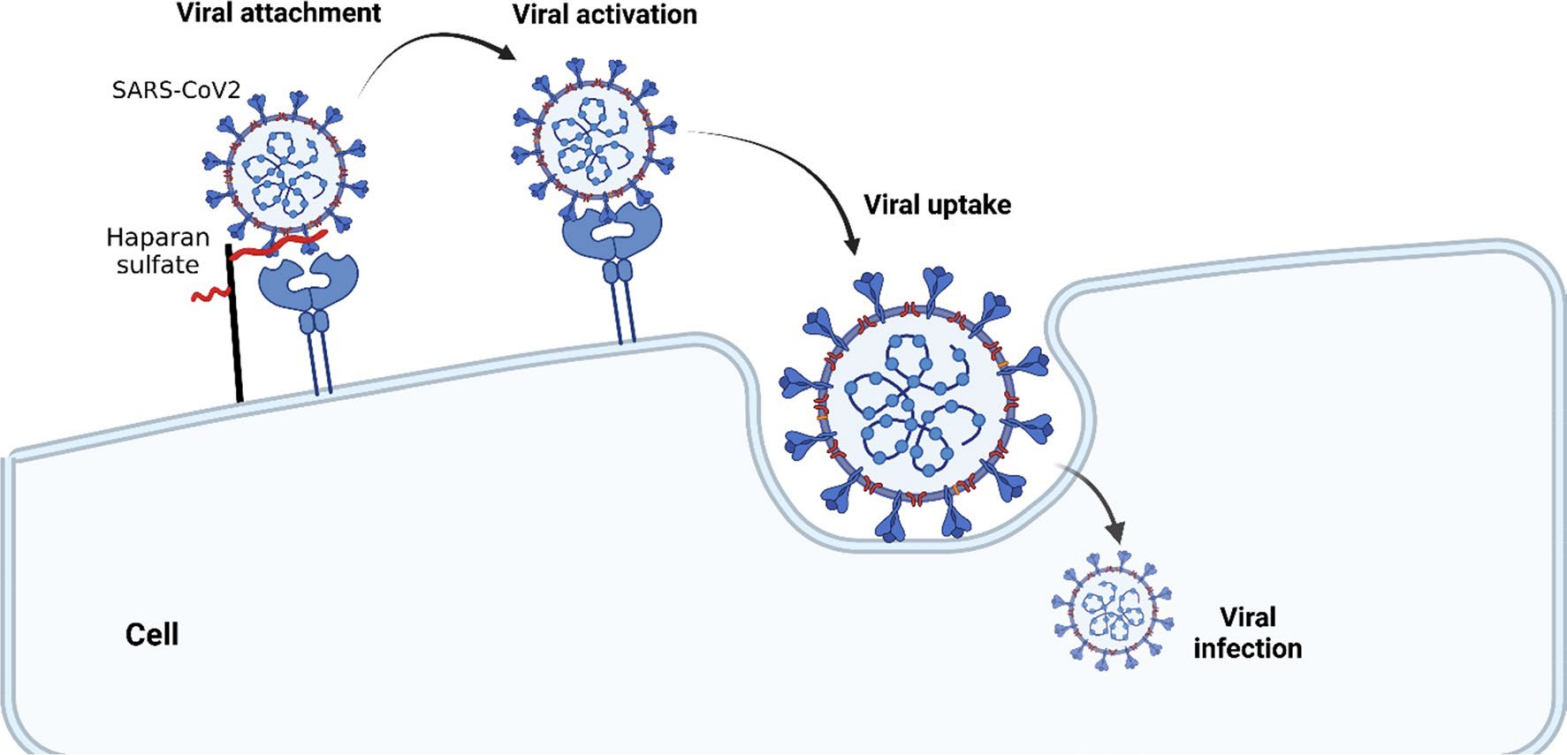
Role of heparan sulfate (HS) in the entry of SARS-CoV-2: HS serves as a co-receptor for spike protein of SARS-CoV-2 to binds angiotensin converting enzyme 2 (ACE2). HS binding compounds may compete with the viral particles for attachment to the HSPGs, thus inhibiting the viral engagement of HS chains and the subsequent access to the ACE2 receptor. Consequently, the viral entry by either fusion or endocytosis may be impaired

Notably, the negatively charged HS can interacts and acts as a receptor for the positively charged viral glycoproteins of different viruses. In this state, the viruses use these interactions to augment their load and increase their chance to bind more entry receptors (Rusnati et al. 2009). Moreover, HS acts as specific receptor for *Orthopoxviruses* including monkeypox virus (MPXV) (Alakunle et al. 2020). Recently, it has been shown that HS severs as a co-receptor for spike protein of severe acute respiratory syndrome coronavirus type 2 (SARS-CoV-2) to binds angiotensin converting enzyme 2 (ACE2) (Fig. 1) (Yu et al. 2021).

In this bargain, due to involvement of HS proteoglycan in both MPXV and SARS-CoV-2 infections, this study aimed to find the potential link between SARS-CoV-2 infection and MPXV infection regarding HPSE level.

## Heparanase in viral infections

HPSE is regarded as a connection between HS and viral infections, since HS represents a first line of interaction between host cells and viral particles. Direct regulation of HS by HPSE increases risk of viral binding and entry (Agelidis and Shukla 2020). As well, expression of HPSE is induced and up-regulated during viral infections resulting in degradation of HS and release of pro-inflammatory cytokines. For example herpes simplex virus (HSV) increases expression of HPSE with subsequent development of inflammatory changes (Agelidis and Shukla 2020). Koganti et al., revealed that higher expression of HPSE induced by HSV infection in mice resulted in worsening ocular symptoms (Gross-Cohen et al. 2021). Similarly, human papilloma viruses (HPV) block p53 which limit HPSE activation and expression. Release of chemokines, cytokines and growth factors in HPV infection is augmented due to degradation of HS by HPSE, thus p53 activators like pyranoside decrease HPSE activation (Hirshoren et al. 2014; Song et al. 2016).

Furthermore, respiratory syncytial virus (RSV) up-regulates expression of HPSE during acute respiratory infection with exaggeration of respiratory inflammation (Tao et al. 2014. Su et al., revealed that HS is involved in the pathogenesis of Japanese encephalitis virus (JEV) (Su et al. 2001). However, highly sulfated dextran, heparin and GAGs inhibit binding and entry of JEV in BHK-21 cell line (Su et al. 2001). Thus, heparin could be effective against development of infection by JEV (Su et al. 2001).

As well, human immune deficiency virus 1 (HIV-1) interacts with HS before to be recognized by CD4, causing augmentation of infection (Connell and Lortat-Jacob 2013). HS acts as co-receptor for binding of HIV-1 to pg120. Thus, HS mimetic may inhibit the interaction between HIV-1 and pg120 (Connell and Lortat-Jacob 2013). HS is also used by many pathogens including plasmodium falciparum, pseudomonas aeruginosa, mycobacterium tuberculosis, borrelia budrdorferi and hepatitis viruses (Spillmann 2001; Liu and Thorp 2002; Vivès et al. 2006; Bartlett and Park 2010; Tiwari et al. 2012). Different studies illustrated that elimination of HS augments cell resistance to the many viral infections. However, soluble HS like heparin and dextran bind the circulating viruses and prevent their binding to the cell membrane HS (Urbinati et al. 2008; Walker et al. 2002). The interaction between HS and viral molecules may cause adaptation changes with increasing affinity to HS (Bear et al. 2006). Taken together, HS increases entry and pathogenesis of viruses but HS derived molecules may have antiviral effects, thus these molecules could be effective therapeutic tools (Hu et al. 2011). These observations suggest that HS plays a critical role in the pathogenesis of viral infections.

## Heparanase and immune response

**Fig. 2:**
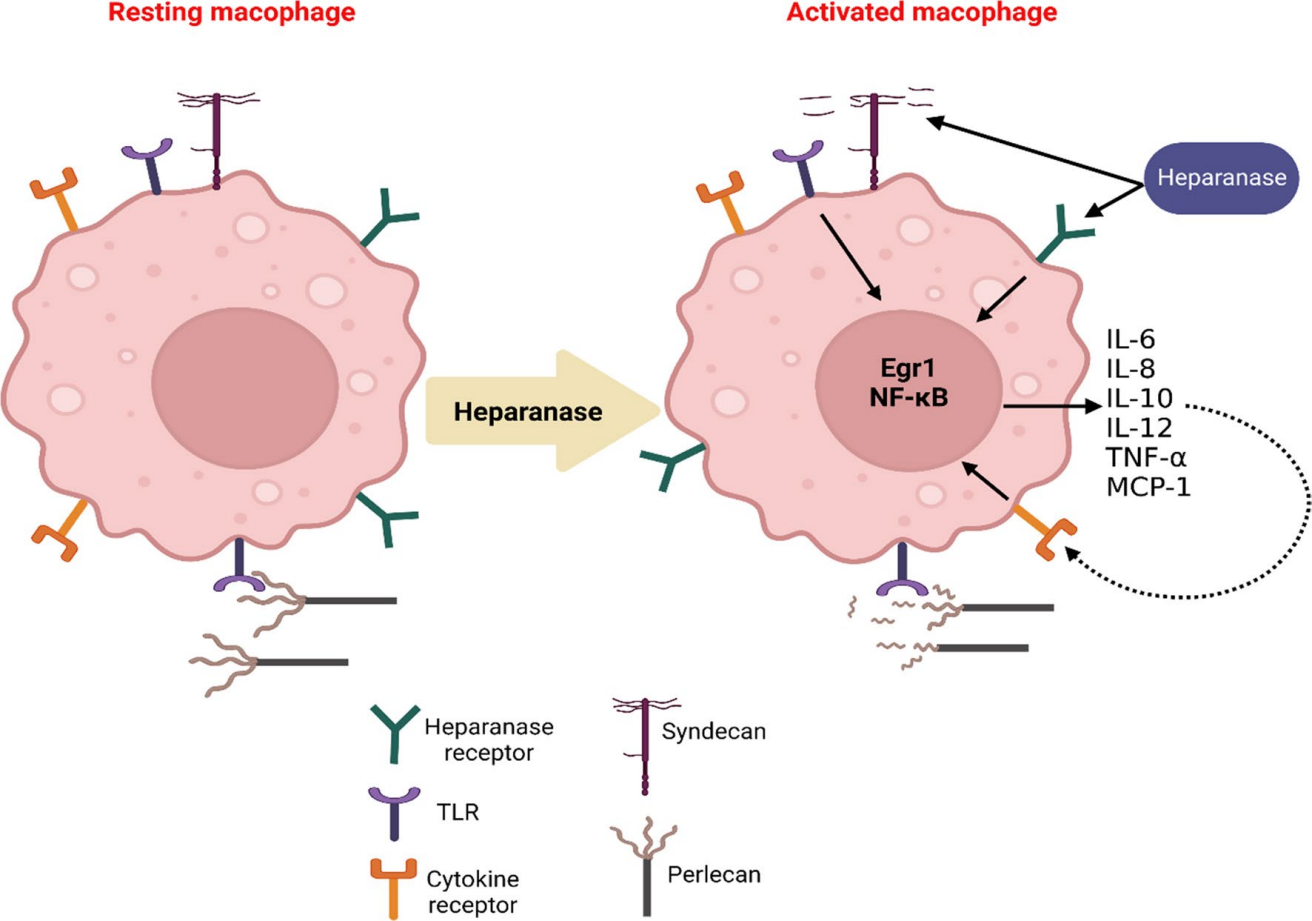
Heparanase (HPSE) and macrophage activation: HPSE activates resting macrophage to be converted to activated macrophage with activation of cytokine receptors. HPSE via HPSE receptors increases expression of Fgr1 and NF-κB which induces expression and release of pro-inflammatory cytokines like IL-6 and IL-2. HPSE and pro-inflammatory cytokines also stimulate the activated macrophage to release more of pro-inflammatory cytokines

HPSE has a crucial role in the activation of macrophage and release of pro-inflammatory cytokines during viral infections (Wagner et al. 2021). Non-enzymatic activation of HPSE promotes cytokine expression, suggesting that HS is not necessary in HPSE-induced macrophage activation (Wagner et al. 2021). Besides, in vitro study demonstrated that macrophage inflammatory protein 2 (MIP-2) can induces activation of HPSE (Wagner et al. 2021). Notably, HPSE increases polarization of macrophage to tumorigenic phenotype (Fig. 2) (Bhattacharya et al. 2020). Furthermore, HS HS fragments cleaved by HPSE induces activation of toll-like receptor 4 (TLR-4) which promote activation of inflammatory signaling pathways including JNK, ERK and p38 trigger some cytokines necessary for macrophage activation (Wagner et al. 2021). Likewise, activation of TLR-4 by HS and HPSE stimulate a series of inflammatory signaling pathways like NF-κB, PI3K and MAPK which provoke macrophage activation and release of pro-inflammatory cytokines (Takeda and Akira 2004). Furthermore, activated macrophages can secret pro-inflammatory cytokines like TNF-α which increase expression of HPSE. As well, activated macrophages release cathepsin L which also activates expression and activation of HPSE with subsequent propagation of hyperinflammation state which promote neovascularization and tumorigenesis (Lerner et al. 2011).

It has been reported that activated T cells can release HPSE which increase binding of T cells to the extracellular matrix proteins (Goldshmidt et al. 2003). This mechanism is depending on presence of β1 integrin and vascular cell adhesion molecule 1 (VCAM-1) on T lymphocytes and endothelial cells respectively (Goldshmidt et al. 2003). Furthermore, HPSE can stimulate activation of natural killer (NK) cells through activation of natural cytotoxic receptors which increase expression of pro-inflammatory cytokines. In this state HPSE has ability to remove HS which act as a co-ligand in preventing activation of NK cells (Mayes et al. 2017).

Notably, HPSE increases activation and release of VEGF which normally sequestered by HS (Elkin et al. 2001). It has been reported that VEGF is activated during viral infections (Fleming et al. 2015). Releasing of VEGF by HPSE-dependent mechanism is occurs via degradation of HS bounded to the VEGF(Elkin et al. 2001). In addition, HPSE encourage release of EGF and TGF-α via activation of ERK and MAPK signaling pathways (Koroglu et al. 2010).

Interestingly, HPSE assists in egress of various viruses like HSV from host immune response by releasing of bounded HS from invading virus. HPSE increases shedding of syndecan from host cell membrane through activation release of matrix metalloproteinase (MMP) (Surviladze et al. 2015). As well, virus binding to HS can be released outside the infected cells without immune recognition (Surviladze et al. 2015). Cleaving of HS by HPSE increases viral release and infectivity, herein pharmacological inhibition of HPSE may reduce viral release and pathogenesis of viral infections (Surviladze et al. 2015). Activation of HPSE during viral infection may increase disease severity by inhibiting release of type 1 interferon which has potent antiviral effect (Agelidis et al. 2017).

These verdicts pointed out that HPSE has important immunological effects through activation of macrophage, T cell and NK cells with stimulation of inflammatory signaling pathways and growth factors during viral infections. These changes may increase disease severity during viral infections. So, targeting of HPSE by specific antagonists may reduce immunological overreaction during viral infections.

## Heparanase in Covid-19

Coronavirus disease 2019 (Covid-19) is an existing pandemic disease caused by severe acute respiratory syndrome coronavirus virus respiratory type 2 (SARS-CoV-2) (Al-Kuraishy et al. 2021a, Al-Kuraishy et al. 2022c). SARS-CoV-2 is a single strand RNA virus from *Betacoronavireadae* family, which has a close-up genetic correspondence with other coronaviruses like bat coronavirus, SARS-CoV and Middle East Respiratory Syndrome coronavirus virus (MERS-CoV) (Al-Kuraishy et al. 2021b,2022c). SARS-CoV-2 was originally appeared in Wuhan, China, led to unrecognized pneumonia named Wuhan pneumonia (Al-Kuraishy et al. 2021c). Further, this virus was renamed as a novel coronavirus virus 2019 (nCov2019). Then the world health organization (WHO) declared this disease as a pandemic and renamed this virus to SARS-CoV-2 (Al-Kuraishy et al. 2021d). Covid-19 is considered as a respiratory disease causing respiratory symptoms similar to the flue like illness characterized by fever, headache, dry cough, dyspnea, myalgia, joint pain, and anosmia (Al-Kuraishy et al. 2021e, Al-Kuraishy et al. [Bibr CR22][Bibr CR14]). Further scrutinized researches exposed that Covid-19 may cause extra-pulmonary manifestations including acute kidney injury, thromboembolic disorders, gastrointestinal and neurological complications (Al-Kuraishy et al. 2020a, Al-Kuraishy et al. 2022, 2021e). Generally, Covid-19 is typically asymptomatic in about 85% of affected patients. Though, 15% of the affected patients presented with severe dyspnea and critical respiratory symptoms due to promulgation of acute lung injury (ALI). Additionally, 5% of Covid-19 patients require hospitalization and intensive care unit (ICU) admission due to progression of acute respiratory distress syndrome (ARDS) (Al-Kuraishy, Al-Gareeb, Qusty, et al. 2021f, Al-Kuraishy et al. 2022). Severely affected Covid-19 patients may necessitate invasive oxygen supplementation and mechanical ventilation (Al-Kuraishy et al. 2021f, Al-Kuraishy et al. 2022g).

Management of Covid-19 patients is mostly supportive and symptomatic alleviate as explicit anti-SARS-CoV-2 was not build up till now in spite of advance of effective vaccines. Noteworthy, numerous repurposed agents like ivermectin, remdesivir, and favipiravir were incorporated in dissimilar therapeutic protocols in the management of Covid-19 (Carlotti et al. 2020, Al-Kuraishy et al. 2022h). Though, these agents did not fashioned to be effectual therapeutic agents in the eradication of SARS-CoV-2, thus prolong searching for novel anti-SARS-CoV-2 agents is a type of brave nowadays (Carlotti et al. 2020).

In SARS-CoV-2 infection, HPSE is increased leading to degradation of endothelial glycocalyx with increment of vascular inflammation and leakage with subsequent development of endothelial dysfunction (ED) a hallmark of Covid-19 (Buijsers et al. 2020). A prospective study involved 48 Covid-19 patients compared to 10 healthy controls illustrated that HPSE and HS serum levels were increased in Covid-19 patients as compared to the controls (Buijsers et al. 2020). Higher HPSE level was linked with Covid-19 severity and ICU admission (Buijsers et al. 2020). Nadir and colleagues found that higher expression of HPSE and HS are correlated with Covid-19 severity (Nadir and Brenner 2021). HPSE is increased in elderly subjects by whom pulmonary HS and other GAGs are reduced with increasing risk of SARS-CoV-2 infection in elderly (Nadir and Brenner 2021). It has been proposed that increasing HPSE in older age individuals enhances degradation of HS in lung and endothelial cells with more risk for development of ARDS and ED in Covid-19 patients (Nadir and Brenner 2021).

**Fig. 3:**
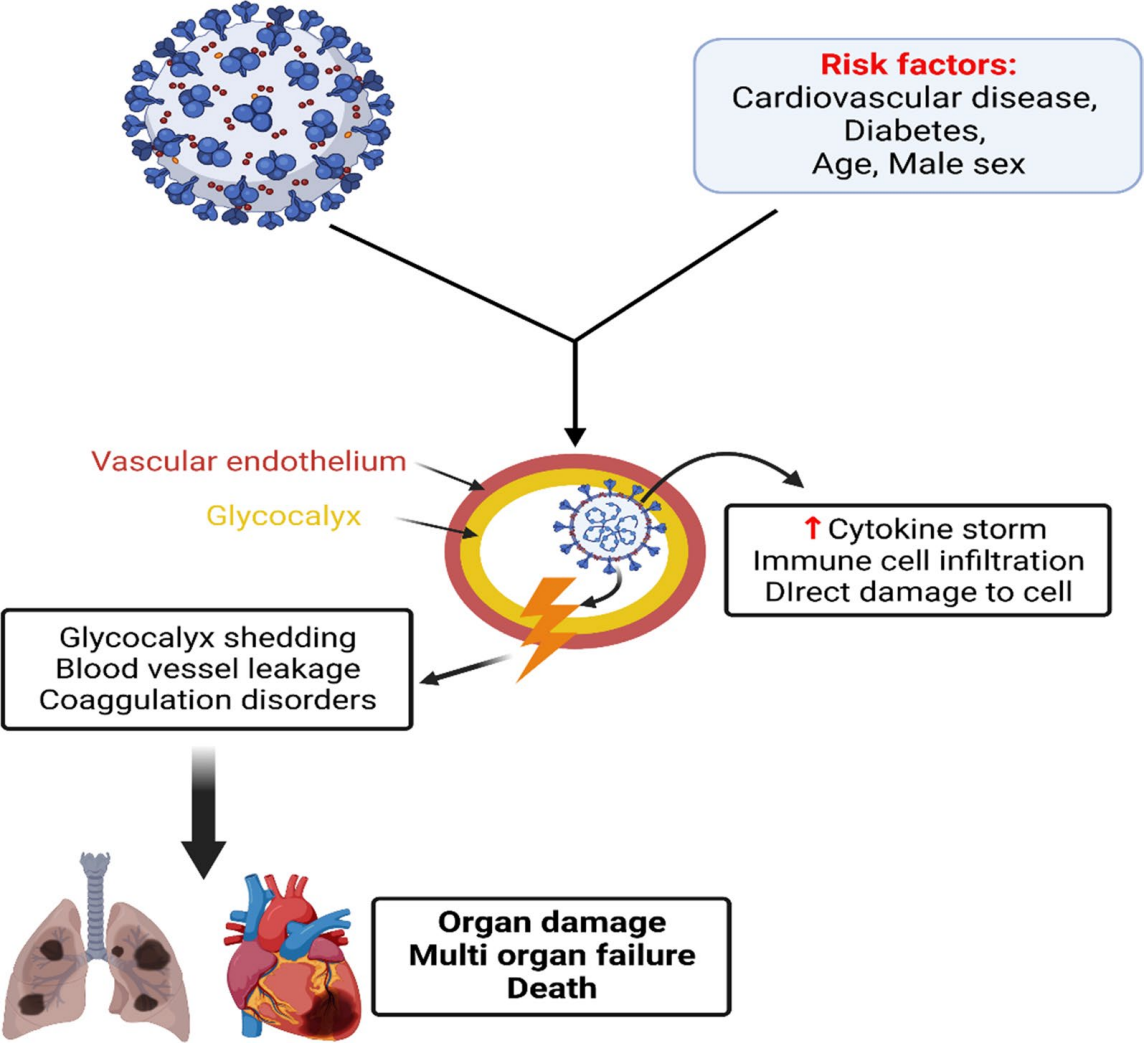
Glycocalyx injury in Covid-19 and systemic complications: Co-morbidities like old age, diabetes and cardiovascular disorders together with SARS-CoV-2 induce glycocalyx injury with development of endothelial dysfunction, blood vessel leakage and coagulation disorders. These changes lead to organ damage, multi-organ failure, and death. Covid-19-induced glycocalyx injury is mediated by cytokine storm, immune cell infiltration, and direct cellular injury

Notably, endothelial glycocalyx which cover the endothelial cells has an important role in maintaining of vascular endothelial homeostasis. Disruption of endothelial glycocalyx is associated with poor clinical outcome in severely affected Covid-19 patients (Stahl et al. 2020). A clinical study included severely affected Covid-19 patients at ICU showed that biomarkers of endothelial glycocalyx injury like HPSE and HS were increased as compared to healthy controls (Stahl et al. 2020). Thus, HPSE inhibitors may reduce development of ED by suppressing injury of endothelial glycocalyx in Covid-19 patients (Fig. 3) (Rus et al. 2022).

Of note, ED is associated with development of Covid-19 complications including ARDS and acute kidney injury (AKI) (Al-Kuraishy et al. 2022). HS is the main GAG present in the endothelial cells glycocalyx. HS due to its negative charge can play an important role in maintaining of charge-dependent endothelial barrier function(Talsma et al. 2018). Degradation of endothelial HS by HPSE impairs glycocalyx barrier and endothelial function. This interaction promotes generation of pro-inflammatory glycocalyx which trigger binding of pro-inflammatory cytokines to the endothelial cells (McDonald et al. 2016). La Riviere and Schmidt observed that injury of pulmonary endothelial glycocalyx during sepsis promotes development of ALI, ARDS and alveolar micro-vascular dysfunction (LaRivière and Schmidt 2018). Biomarkers of glycocalyx injury like HS and syndecanes are increased during sepsis-induced endothelial dysfunction (Iba and Levy 2019). In SARS-CoV-2 infection degradation of HS by HPSE induces activation of bradykinin with development of pulmonary inflammation and ARDS. Normally, HS inhibits bradykinin activation so over-activation of HPSE in Covid-19 with degradation of HS provokes activation of bradykinin (Liu et al. 2020). Besides, deregulation of renin-angiotensin system (RAS) due to downregulation of ACE2 increases circulating level of angiotensin II (AngII) with subsequent development and propagation of endothelial dysfunction (Al-Kuraishy et al. 2020b, Al-Kuraishy, Al-Niemi, et al. 2020; Al-Kuraishy, Al-Gareeb, Alzahrani, Alexiou, et al. 2021). Different studies showed that AngII had ability to induce expression of HPSE and endothelin-1 (ET-1) which also promote HPSE expression (Van den Hoven et al. 2009; Hong et al. 2004). As well, AngII enhances bradykinin activation and development of ED (Hasan et al. 2021).

Over-activation of HPSE and releases of HS in severe SARS-CoV-2 infection induce activation of macrophage through TLR-4 with subsequent release of pro-inflammatory cytokines (Buijsers et al. 2020). Free HS acts as a pro-inflammatory agent provokes immune cells stimulation (Buijsers et al. 2020). In turn, exaggerated immune response and hypercytokinemia in severe SARS-CoV-2 infection promote HPSE expression in a positive feed-back loop (Buijsers et al. 2020, Al-kuraishy et al. 2022). Therefore, inhibition of HPSE by low molecular weight heparin (LMWH) may reduce endothelial injury and exaggeration of immune response by inhibiting release of pro-inflammatory cytokines (Shen et al. 2022). In addition, prophylactic use of LMWH in Covid-19 patients can decrease HPSE expression and activation (Shen et al. 2022). However, HPSE level remains elevated in severely affected Covid-19 patients despite use of LMWH (Buijsers et al. 2020). This feature suggest that early use of LMWH may reduce HPSE level by preventing endothelial glycocalx injury but use of LMWH in the late phase of Covid-19 may not reduce HPSE level as it released in the early stage mainly in severely affected Covid-19 patients.

These observations proposed that HPSE and HS are highly deranged in Covid-19 leading to activation release of pro-inflammatory cytokines and increase risk for development of cytokine storm. As well, HPSE and HS serum levels could be potential biomarkers for assessment of Covid-19 severity.

## Heparanase and coagulopathy in Covid-19

**Fig. 4:**
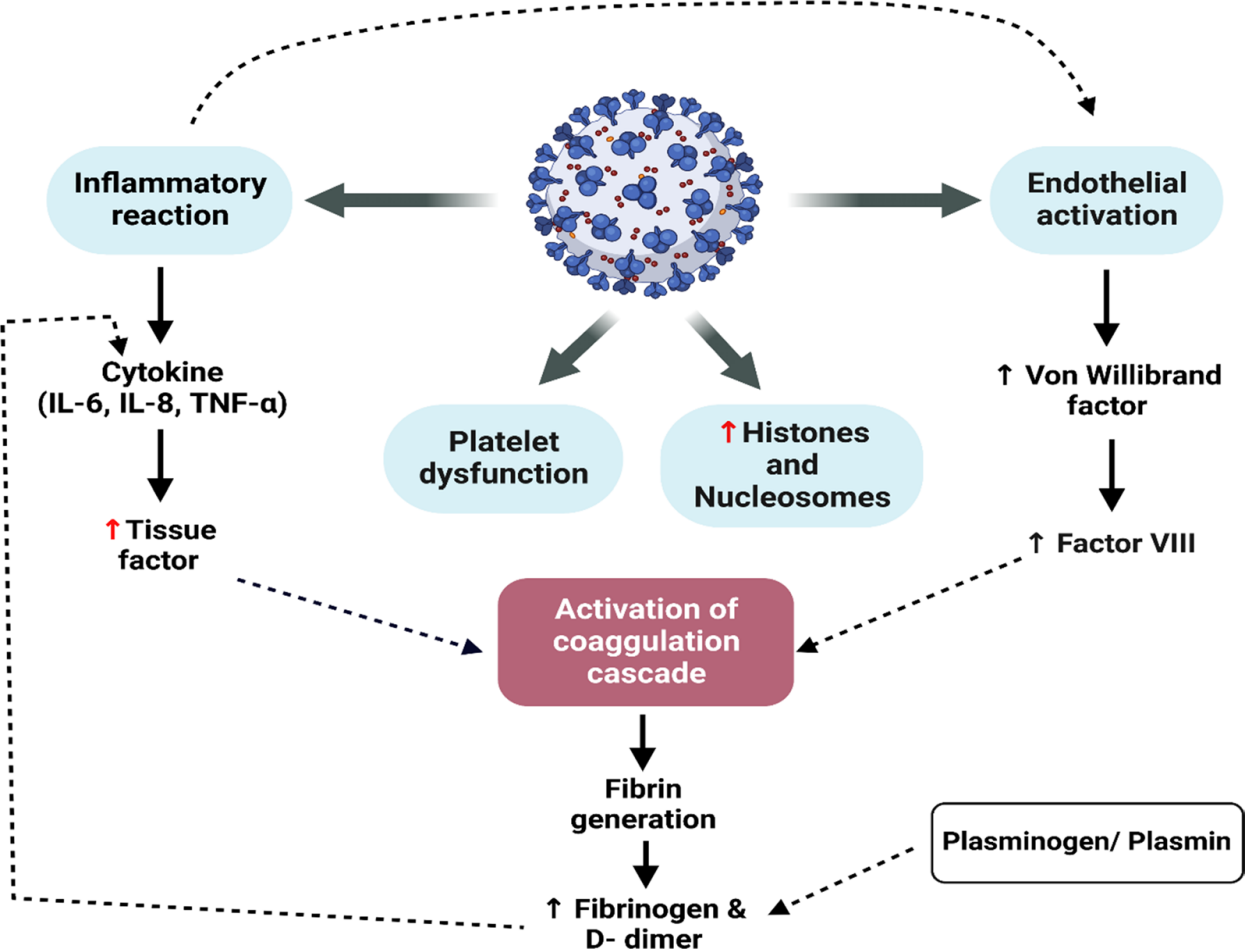
Coagulopathy in Covid-19: SARS-CoV-2 infection leads to inflammatory reactions, endothelial dysfunction, platelet dysfunction and increase release of histone and nucleosomes. These changes trigger activation of coagulation cascade with thrombotic disorders and increase D-dimer. Fibrinogen and D-dimer activate release of pro-inflammatory cytokines

It has been shown that severe SARS-CoV-2 infection is associated with development of thrombotic leading to pulmonary micro-thrombosis and development of ARDS. As well, Covid-19 may induce progression of systemic thrombosis and disseminated intravascular coagulopathy (DIC). These changes are developed due to SARS-CoV-2 infection-induced ED [Fig. 4]. (Asakura and Ogawa 2021; Al-Kuraishy et al. 2021e).

Activation of HS and HPSE may be implicated in the pathogenesis of coagulopathy. Normally, tissue factor (TF) is the chief initiator of coagulation; it is not expressed by endothelial cells under physiological condition (Reeves et al. 2018). Though, during systemic inflammation and dysregulation of RAS, the expression of TF by the endothelial cells is augmented (Reeves et al. 2018). TF induces coagulation cascade as it act as a receptor for clotting factor VII (Zhao et al. 2021). TF is modulated by many endogenous proteinases expressed on the endothelial cells, called TF pathway inhibitor (TFPI) (Zhao et al. 2021). Therefore, activation of TF and TFPI trigger development of thrombosis in severe viral infections and cancer (Zhao et al. 2021). Over-expression of HPSE and degradation of HS can cause coagulopathy through induction expression of TF and TFPI (Kinaneh et al. 2021). In addition, HPSE facilitates the interaction between TF and activated VII with consequent escalation of thrombosis (Kinaneh et al. 2021). As well, activated platelets produce and release HPSE with further thrombotic development (Kinaneh et al. 2021).

Of note, HS enhances SARS-CoV-2 and other coronavirus infections to bind ACE2 with further viral entry (Hao et al. 2021). Likewise; SARS-CoV-2 induces expression of HPSE with more degradation of HS in endothelial glycocalyx (Buijsers et al. 2020). Interestingly, up-regulation of HPSE in SARS-CoV-2 infection could the potential cause for development of coagulopathy and fatal complications (Buijsers et al. 2020). Thus, administration of LMWH in severely affected Covid-19 patients may decrease risk of thrombotic complications by inhibiting HPSE expression and activation of TF/TFIP axis (Grandone et al. 2021). Furthermore, HS mimetic compounds like pixatimod which is a potent inhibitor of HPSE might be of value in the management of Covid-19 by inhibiting HPSE-induced inflammation and coagulopathy (Guimond et al. 2022).

These findings indicate that activated HS/HPSE in SARS-CoV-2 infection may participate in the progression of coagulopathy. Thus, LMWH and HS mimetic can reduce HPSE in SARS-CoV-2 infection with further attenuation of thrombotic complications.

## Heparanase and cytokine storm in Covid-19

Cytokine storm (CS) in Covid-19 is propagated due to complex immunological response characterized by over-activation of T1h immune response and weak IFN response with subsequent stimulation of inflammatory signaling pathways and excessive release of pro-inflammatory cytokines (Hu et al. 2021). Development of CS is correlated with Covid-19 severity due to hypercytokinemia-induced organ injury (Hu et al. 2021). Remarkably, activation of HPSE with subsequent release of pro-inflammatory HS promotes release of pro-inflammatory cytokines with development of CS during sepsis (Vlodavsky et al. 2021). Of note, activated HPSE increases activation and polarization of macrophages, T cells and NK cells with induction expression of TLR4 (Wagner et al. 2021) (Bhattacharya et al. 2020; Goldshmidt et al. 2003; Mayes et al. 2017). Besides, activation of HPSE promotes expression of inflammatory signaling pathways including NF-κB, PI3K and MAPK which increase macrophage activation and release of pro-inflammatory cytokines (Takeda and Akira 2004). In addition, activated macrophages can secret pro-inflammatory cytokines like TNF-α which increase expression of HPSE (Takeda and Akira 2004). Additionally activated macrophages release cathepsin L which also increases HPSE expression with propagation of hyperinflammation state (Lerner et al. 2011). Different studies observed that higher expression of inflammatory signaling pathways in severe Covid-19 may increase risk for development of CS by inducing release of pro-inflammatory cytokines and chemokines (Kim et al. 2021; Yang et al. 2021).

These judgements proposed that higher HPSE expression in Covid-19 may linked with development of CS and disease severity. Targeting of HPSE in the early stage of Covid-19 may reduce propagation of CS and associated life-threatening complications.

## Heparanase in Monkeypox

Monkeypox (MPX) is one of common zoonotic disease caused by a double strand DNA MPX virus (MPXV) belonging of *Orthopoxvirus* genus/ *Poxviridae* family. MPXV is extremely pathogenic for human subsequent to the eradication of smallpox in 1980 (Alkhalil et al. 2010; Di Giulio and Eckburg 2004). The natural host of MPXV is not certainly identified, though it infects a wide-spectrum of animal and mammalian species. MPX is mostly endemic in Democratic Republic of Congo (DRC) and some area of Ivory Coast (Alkhalil et al. 2010; Di Giulio and Eckburg 2004). Particularly, there are two clades of MPXV that vary clinically and epidemiologically. Central African clade (Congo Basin clade) characterized by elevated case fatality rate (CFR) about 11% with confirmed person-to-person transmission. Though, West African clade is characterized by low CFR about 1% without person- to- person transmission (Likos et al. 2005).

MPX was initially documented in 1958 as smallpox-like disease in the laboratory monkeys in Denmark by Preben von Magnus (Xiang and White 2022). The first reported case of human MPX was in 1970, and on 1972 a case of human MPX was recognized a 9-month neonate in DRC (Xiang and White 2022). A sum number of 50 reported cases of human MPX were established from 1970 to 1979, two thirds of these cases being from DRC (Breman et al. 1980). Meyer et al., reported that by end of 1986 more than 400 cases of human MPX characterized by 10% CFR were identified in West and Central Africa that was design as a first outbreak (Meyer et al. 2002). The second outbreak of human MPX was identified in DRC in a period between 1996 and 1997 (Qiao et al. 2020). In a period from 1991 to 1999 a 511 reported cases of human MPX were celebrated in DRC (Meyer et al. 2002).

The clinical picture of human MPX is highly identical to that of smallpox, nevertheless early lymphadenopathy in human MPX is the distinguishing sign not present in smallpox. The incubation period is 1–3 weeks, fever, headache, joint pain, myalgia and nausea for about 3 days (Minhaj et al. 2022). Skin lesions which appear 1–3 days subsequent fever and lymphadenopathy are typically appearing at the same time on the face and periphery (Weinstein et al. 2005) (Adler et al. 2022). Lymphadenopathy characterized by lymph node enlargement mainly in the neck, groin, and submandibular area. The skin lesions cover all the body in severe cases. The skin lesions started as a small flat spot which become small bumps (papules) which later filled with clear fluid and then with yellow fluid, sometimes merge to form large lesions. The lesions progress in the same time similar to that of smallpox, following healing the lesions leaves pale marks which finally become darks (Adalja and Inglesby 2022).

Transmission of MPXV is frequently occurring through direct contact and respiratory droplets. Sexual contact with animals could be a probable cause (Organization 2022). The skin rashes go in various stages before forming scab which finally fall out. Two third of patients have skin lesions on the palms and soles (Organization 2022). Diagnosis of human MPX is done depending on the clinical features and history for contacts with animals. Polymerase chain reaction (PCR) of samples from skin lesions is definitive for the final diagnosis. Blood PCR is not a definitive since MPXV is no longer persisting in the blood (Organization 2022). Human MPX in severe conditions may lead to various complications including bronchopneumonia, acute respiratory distress, sepsis, gastrointestinal complications, dehydration, encephalitis, and visual loss due to involvement of cornea. Human MPX may be misdiagnosed with chickenpox, smallpox, anthrax and HIV-induced skin lesion (Adalja and Inglesby 2022).

## Pathogenesis and immunological response of MPXV

**Fig. 5:**
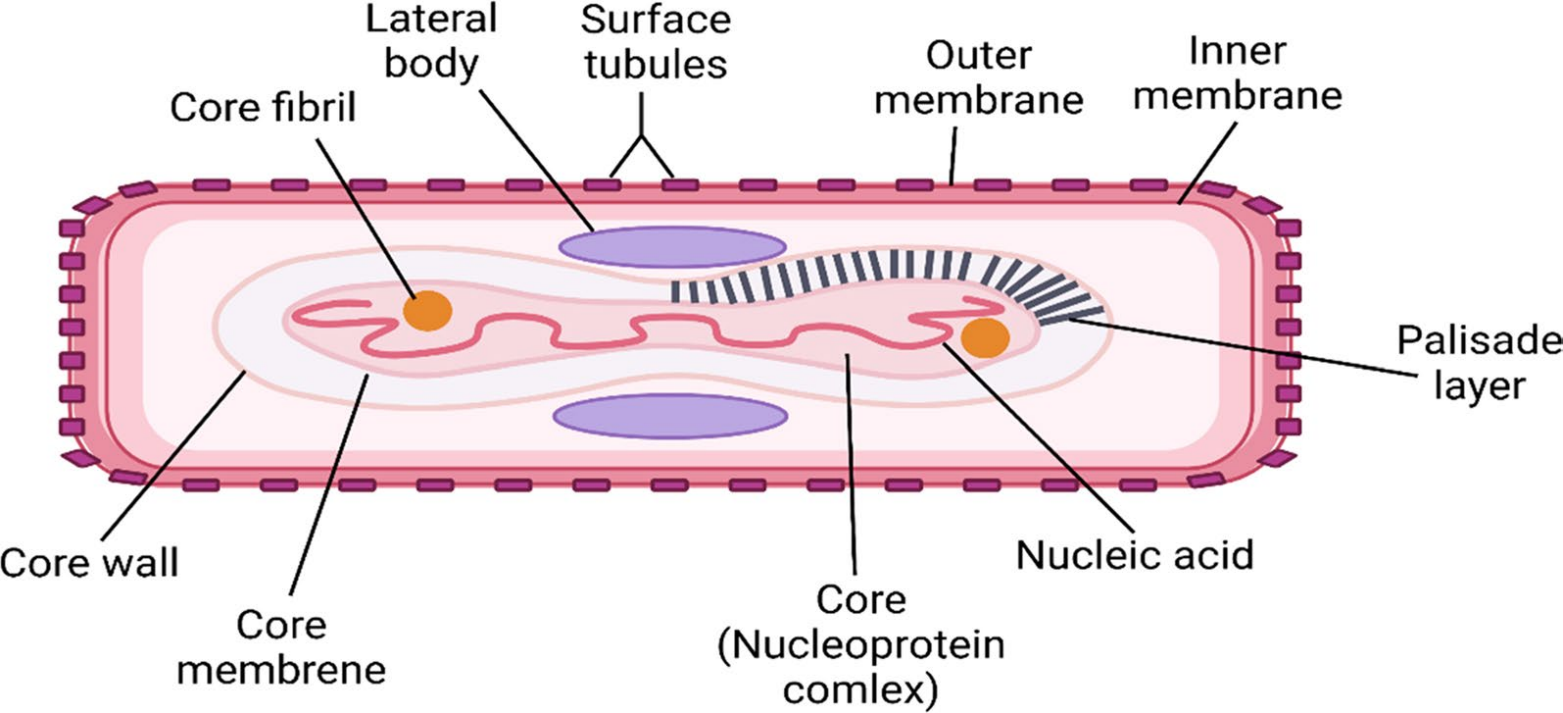
Structure of monkeypox virus: Human MPX is an enveloped dsDNA MPXV which has 250 nm width and 170–250 kb in size of DNA genome. MPXV consist of surface tubules, outer envelope of extracellular virion, lateral bodies, plasid layer, core fibrils, and outer membrane of intracellular and extracellular virions

Human MPX is caused by an enveloped dsDNA MPXV which has 250 nm width and 170–250 kb in size of DNA genome. MPXV consist of surface tubules, outer envelope of extracellular virion, lateral bodies, plasid layer, core fibrils, and outer membrane of intracellular and extracellular virions (Fig. 5).

MPXV enters the mucous membrane of mouth, eye and respiratory tracts (Guarner et al. 2004). Similar to the other *Orthopoxviruses,* entry of MPXV into host cells is achieved by binding to the glycosaminoglycan (GAGs) on the cell membrane, which mediate viral endocytosis (Guarner et al. 2004). In the cytoplasm the viral core and genomes are released with succeeding replication of viral proteins by viral DNA polymerase. Indeed, viral structural proteins are produced within 48 h post-infection with subsequent assembly into mature virions in the Golgi apparatus. From Golgi apparatus, the mature virions are transported by microtubules to the plasma membrane and released outside the infected cells to infect other cells in the same manner (Likos et al. 2005).

There are four types of GAGs including HS, chondriotin/dermatan sulfate, keratin sulfate and hayluronic acid (Bartlett and Park 2010; Gandhi and Mancera 2008; Sodhi and Panitch 2020). GAGs are complex carbohydrate ubiquitously expressed on cell surface and extracellular matrix. The interactions between GAGs and microbial pathogens represent a defense line against invasion (Lin et al. 2020). Several pathogens including MPXV induce release of GAGs with formation of soluble GAGs which coat the pathogen to escape immune detection (Akhtar and Shukla 2009; Hughes et al. 2014). HS together with sialic acid are highly expressed in dermal and epidermal cells that enhance binding and entry of MPXV to the skin (Hughes et al. 2014).

In MPXV like other *Orthopoxviruses* there is activation of genes concerned in activation expression of pro-inflammatory cytokines and chemokines like IL-6 and CCL-2 respectively (Bourquain et al. 2013). As well, MAPK and ERK are activated by MPXV which encourage viral entry, viral replication and augment expression of viral proteins needed for viral replication (DuShane and Maginnis 2019). Furthermore, heat shock protein 1 (HSP-1) is necessary for replication of MPXV so it highly induced during infection with MPXV (Filone et al. 2014).

Notably, MPXV induces activation of NF-κB through suppression of signal transducer and activator of transcription (STAT) which has antiviral effects (Filone et al. 2014). Besides, MPXV activates NK cells to release interferon gamma (INF-γ) and TNF-α which stimulate Th1 immune response (Filone et al. 2014). Overall, due to selective tropism of lymphoid tissues, MPXV can induce lymphopenia and lymphadenopathy (Townsend et al. 2013). Inhibition of CD4 and CD8 as well as maintaining of major histocompatibility complex 1 (MHC1) could be the probable mechanism for immune evasion of MPXV (Hammarlund et al. 2008). MPXV can evade the immune response through release of viroceptors and virokines which are like to the host cytokines. These annotations may clarify the immunosuppressive effect of MPXV as evident by reduction of T lymphocytes in MPX (Hammarlund et al. 2008). Consequently, the immune response unlike other viral infections is complex and need further attention. These verdicts may explain the immunosuppressive effect of MPXV as evident by reduction of T lymphocytes in MPX (Townsend et al. 2013; Hammarlund et al. 2008).

Different studies showed that binding of MPXV to the HS may induce activation of HPSE and release of pro-inflammatory cytokines (Khanna et al. 2017; Bhatt et al. 2021; Kindrachuk et al. 2012). In severe MPXV infection, activation of inflammatory signaling pathways with release of pro-inflammatory cytokines may lead to the development of CS. It has been shown that MPX may causes fatal complications like sepsis, bronchopneumonia and ALI (Stittelaar et al. 2005) which might develop due to propagation of CS. Stanford et al. observed that severity of MPX and smallpox was related to the direct cytopathic effect and immunopathological changes due to release of immunomodulatory molecules. Invasion of injured tissues by immune and inflammatory cells due to exaggerated immune response may cause extensive tissue damage due to propagation of CS (Stanford et al. 2007).

## The potential link between MPX and Covid-19

In both Covid-19 and MPX, lymphopenia is developed by different mechanisms. Direct injury of lymphocyte by SARS-CoV-2, lymphocyte exhaustion and sequestration of lymphocytes in the lymphoid tissues could be the possible cause of lymphopenia in Covid-19 (Fathi and Rezaei 2020). Similarly, exaggeration immune response and development of CS may induce lymphocyte apoptosis inhibit bone marrow proliferation and production of lymphocytes (Fathi and Rezaei 2020). Of note, lymphopenia is associated with Covid-19 severity due to loss of anti-inflammatory function of lymphocytes (Fathi and Rezaei 2020). A systematic review and meta-analysis from 24 studies included 3099 Covid-19 patients showed that lymphopenia at time of admission is linked with poor clinical outcomes and high mortality (Huang and Pranata 2020). In MPX, lymphopenia is a characteristic pathognomic feature due to lymphotropism of MPXV with inhibition release of lymphocytes (Mucker et al. 2015). As well, abnormal immune response and hypercytokinemia in MPX exhausts circulating lymphocytes with development of lymphopenia (Stanford et al. 2007).

Furthermore, over-expression of HPSE in both Covid-19 and MPX with higher immune response may induce propagation of lymphopenia (Digre et al. 2017). Of note, inflammatory signaling pathways and pro-inflammatory cytokines are exaggerated in both Covid-19 and MPX leading to hyperinflammation and development of CS (Buijsers et al. 2020; DuShane and Maginnis 2019). Interestingly, degradation and release of pro-inflammatory HS is triggered by HPSE. SARS-CoV-2 and MPXV are implicated to induce HPSE expression and dissociation of HS from cell membrane GAGs (Buijsers et al. 2020; Lin et al. 2020). Pro-inflammatory HS and activated HPSE promote release of pro-inflammatory cytokines which also trigger stimulation of HPSE in a positive loop manner in SARS-CoV-2 and MPXV infections (Kindrachuk et al. 2012; Yang et al. 2021; Kim et al. 2021).

Up-regulation of HPSE in SARS-CoV-2 infection might be the potential cause for progression of coagulopathy (Buijsers et al.^2020^). Though, HPSE expression and development of coagulopathy was not precisely reported in MPX (Hutson and Damon 2010). The underlying mechanism of coagulopathy and thrombotic events in Covid-19 and MPX could in part relate to HPSE expression. Thus, administration of LMWH in severely affected Covid-19 or MPX patients may reduce risk of thrombotic complications by inhibiting HPSE expression and activation of TF/TFIP axis (Hutson and Damon 2010) (Grandone et al. 2021). What’s more, HS mimetic compounds like pixatimod which is a potent inhibitor of HPSE could be of value in the management of Covid-19 and MPX by inhibiting HPSE-induced inflammation and coagulopathy (Hutson and Damon 2010; Guimond et al. 2022).

**Fig. 6:**
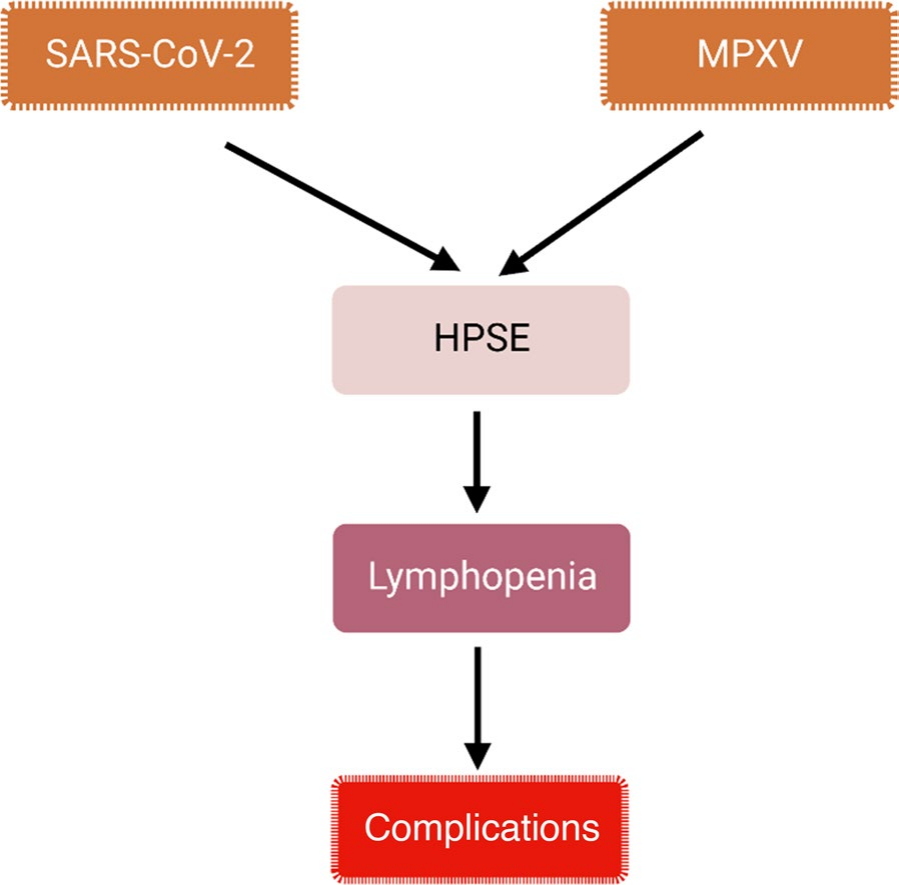
The possible link between SARS-CoV-2 and MPXV infections: SARS-CoV-2 and MPXV infections activate heparanase (HPSE) leading to lymphpenia which cause complications

These findings and observations proposed that HPSE expression which involved in the progression hyperinflammation, CS, and coagulopathy could be the potential link between Covid-19 and MPX (Fig. 6). Targeting of HPSE by treatment with LMWH in severely affected Covid-19 or MPX patients may reduce risk of thrombotic and inflammatory complications. In addition, MPX-induced immunosuppressant effects (Hammarlund et al. 2008) may increase risk of SARS-CoV-2 infection. Co-infection of MPX with SARS-CoV-2 may occur as we facing many outbreaks of MPX cases during Covid-19 pandemic. Further studies are required to find the magic link between MPX and Covid-19.

The present review had many limitations like rareness of clinical studies concerning role MPX in propagation of thrombotic events and CS which are the hallmark of Covid-19. As well, HS level and HPSE activity in relation to pro-inflammatory cytokines and inflammatory signaling pathways were not listed from previous studies in both MPX and Covid-19. Though the present review suggests that HPSE could be the latent link between MPX and Covid-19.

## Conclusions

HPSE is an endoglycosidase cleaves heparin sulfate (HS) and this contributes to the degradation and remodeling of the extracellular matrix. HS cleaved by HPSE induces activation of autophagy and formation of autophagosomes which facilitate binding of HPSE to the HS and consequent release of growth factors and pro-inflammatory cytokines. The interface between HPSE and HS triggers releases of chemokines and cytokines which influence inflammatory response and cell signaling pathways with propagation of hyperinflammation, CS, and thrombotic events. HPSE expression is induced by both SARS-CoV-2 and MPXV in severely affected patients leading to induction release of pro-inflammatory cytokines, endothelial dysfunction, and coagulopathy. Co-infection of MPX with SARS-CoV-2 may occur as we facing many outbreaks of MPX cases during Covid-19 pandemic. Therefore, targeting of HPSE by specific inhibitors like LMWH may reduce risk of complications in both SARS-CoV-2 and MPXV infections. Taken together, HPSE could be a potential link between MPX with SARS-CoV-2 in Covid-19 era. In this regards, various experimental, preclinical, and clinical studies are warranted to explore and confirm the possible role in the pathogenesis of SARS-CoV-2 and MPXV infections.

## Data Availability

All generated data are included in this manuscript.
